# Phytopharmacological Strategies in the Management of Type 2 Diabetes *Mellitus*

**DOI:** 10.3390/foods9030271

**Published:** 2020-03-02

**Authors:** Ana M. Duarte, Maria P. Guarino, Sónia Barroso, Maria M. Gil

**Affiliations:** 1 MARE—Marine and Environmental Sciences Center, Politécnico de Leiria, 2520-630 Peniche, Portugal; ana.c.duarte@ipleiria.pt (A.M.D.);; 2 ciTechCare—Center for Innovative Care and Health Technology, Politécnico de Leiria, 2410-541 Leiria, Portugal; maria.guarino@ipleiria.pt

**Keywords:** type 2 diabetes mellitus, phytopharmacology, dipeptidyl peptidase-4 (DPP4), alpha-amylase, alpha-glucosidase, lipase, protein tyrosine phosphatase 1B (PTP1B)

## Abstract

Type 2 Diabetes *Mellitus* (T2DM) is a chronic disease which corresponds to 90% of the worldwide cases of diabetes, mainly due to epigenetic factors such as unhealthy lifestyles. First line therapeutic approaches are based on lifestyle changes, most of the time complemented with medication mostly associated with several side effects and high costs. As a result, the scientific community is constantly working for the discovery and development of natural therapeutic strategies that provide lower financial impact and minimize side effects. This review focus on these nature-based therapeutic strategies for prevention and control of T2DM, with a special emphasis on natural compounds that present pharmacological activity as dipeptidyl peptidase-4 (DPP4), alpha-amylase, alpha-glucosidase, lipase, and protein tyrosine phosphatase 1B (PTP1B) inhibitors.

## 1. Introduction

**Figure 1:**
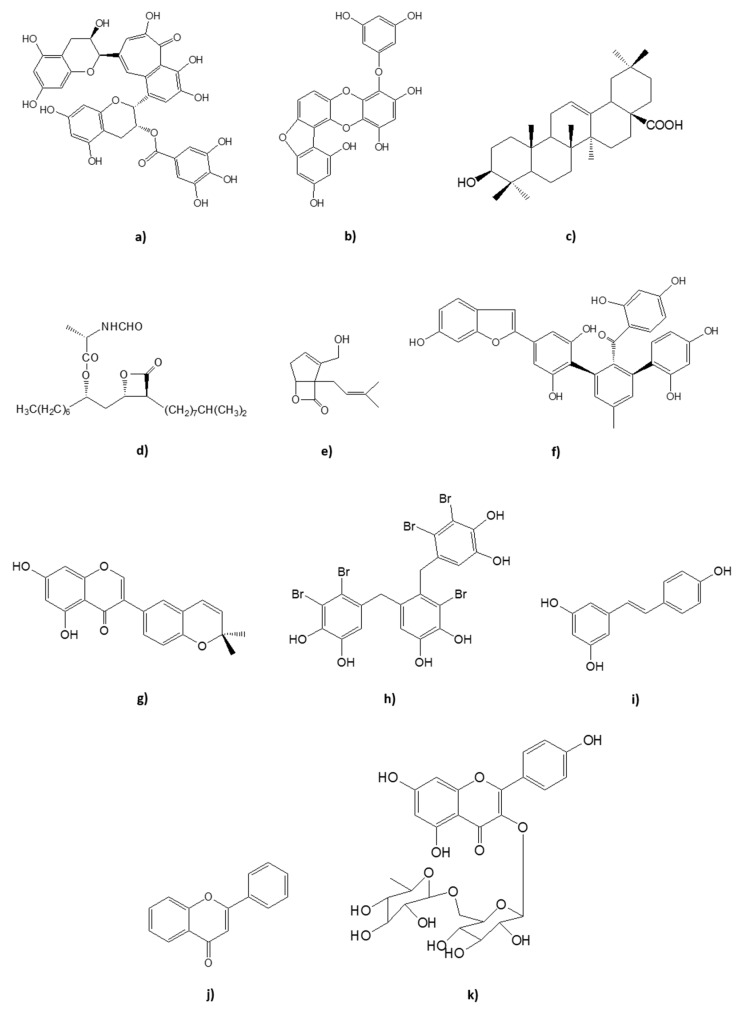
Examples of chemical structures of some of the natural compounds with inhibitory activity against target enzymes in the prevention and control of T2DM. (**a**) theaflavin-3-gallate; (**b**) fucofuroeckol A; (**c**) triterpene oleanolic acid; (**d**) panclicins A; (**e**) percyquinnin; (**f**) mulberrofurans J; (**g**) isoderrone; (**h**) 3-bromo-4,5-Bis-(2,3-dibromo-4,5-dihydroxybenzyl) pyrocatechol; (**i**) resveratrol; (**j**) flavone; (**k**) kaempferol diglycoside.

Diabetes *mellitus* (DM) is a chronic disease characterized by excessive concentration of sugar glucose in the bloodstream, a pathophysiological sign termed: hyperglycemia [1]. There are two main types of DM: type 1 (T1DM) caused by the absence of insulin production due to auto-immune mediated loss of pancreatic β-cells and type 2 (T2DM), which results from the deficient action of insulin, triggering the aberrant synthesis of hepatic glucose, secretion deviations, and insulin resistance in target tissues (liver, muscle, and adipose tissue), with consequent progressive deterioration of pancreatic β-cells functions [1,2,3]. Patients with T2DM are not insulin dependent, unlike those with T1DM, as long as lifestyle interventions and oral hypoglycemic agents are sufficient for effective glycemic control [1,3,4]. Accounting for about 90% of the worldwide cases of DM, and the sixth leading cause of disability, T2DM is clinically detected mainly by the 3 Ps: polyuria, polydipsia, and polyphagia, as well as body weight loss, distorted vision, and fatigue [1,3,4,5,6,7]. The disease can be attributed, on the one hand, to behavioral/environmental factors, and, on the other hand, to not fully understood genetic factors with an influence on β-cells [2,7,8,9]. Nevertheless, the main risk factors for the development of T2DM are oxidative stress, lack of exercise, obesity, and unhealthy diet [2,9]. Inadequate glycemic control can lead to an array of microvascular (e.g., retinopathy, nephropathy, neuropathy) and macrovascular (e.g., cardiovascular diseases such as stroke and heart attack) complications [10]. Thus, it is fundamental to develop effective strategies to restore and maintain blood glucose homeostasis. The aim of this review is to summarize some of the natural therapeutic strategies for prevention and control of T2DM, with a special emphasis on natural compounds that present pharmacological inhibitory activity against dipeptidyl peptidase-4 (DPP4), alpha-amylase, alpha-glucosidase, lipase, and protein tyrosine phosphatase 1B (PTP1B). These natural inhibitors include several classes of compounds such as bromophenols, phlorotannins, sterols, terpenes, stilbenoids, flavonoids, furans, catechols, and fungal metabolites, among others. The structures of some of the natural compounds mentioned across this review are represented in Figure 1.

## 2. Conservative Treatment

The treatment of T2DM safeguards patient-centered therapeutic individualization and is initiated by the alteration of the individual lifestyle, counterworking sedentarism, and obesity through the increase of physical activity and adoption of a balanced diet [11]. However, with progressive decline of pancreatic β-cells function, medication is required generally for extended periods of time [1,11,12,13]. The pharmacologic therapies are mainly based on increasing insulin availability either by direct administration of insulin or via agents promoting insulin secretion, improving insulin sensitivity, delaying gastrointestinal absorption of carbohydrates, and/or increasing glucose excretion [14]. The administration of insulin allows glycemic control, but is related to weight gain due to an increase in body fat mass, especially abdominal obesity, with consequent increase in insulin resistance, as well as episodes of hypoglycemia when the treatment is not performed properly [14]. 

### Lifestyle Interventions: Diet and Physical Activity

Diet influences body weight, glucose, and insulin homeostasis being recognized as a risk factor for the development of T2DM [15,16]. In fact, there are several studies that verify the capacity of prevention and control of metabolic diseases by the food or by specific substances in the diet [16]. There is unanimity on the importance of body weight control, reduction of energy intake coupled with exercise, and healthy diet with low intake of processed foods (rich on refined sugars and flour) and high consumption of whole grains, fiber, polyunsaturated fatty acids, fruits, vegetables, and low-fat dairy products for the control and prevention of T2DM [2,9,16].

Processed red meat belongs to the group of foods to be avoided by the patient with T2DM, although the effect of unprocessed red meat on the pathology is not fully known [16]. The group of forbidden foods for those with T2DM also includes refined grains and sugars (high glycemic index). Preference should be given to the consumption of whole grains (low glycemic index), and, above all, fiber, with a higher consumption being recommended for T2DM patients (50 g per day) than for healthy individuals (30 g per day) [1,16]. Dietary fiber derives from plants and is not hydrolyzable by human digestive enzymes, but is digested by intestinal microflora. Dietary fibers are divided into soluble (e.g., β-glucans, pectins and some hemicelluloses) and insoluble (e.g., cellulose, some hemicelluloses and lignin) [17]. With the exception of lignin, the set of soluble and insoluble fiber, it is called non-starch polysaccharides (NSPs) [17]. When NSPs are ingested and mixed with water, they form a dispersion with an increase in the viscosity of the bolus, reducing the diffusion of digestive enzymes and promoting the sense of satiety, resulting in the fight against obesity, as well as prevention and control of T2DM [12].

On the other hand, there are foods and eating habits whose effect on T2DM remains a case study. Low-calorie and low-carbohydrate diets have beneficial effects on T2DM control, but there is no consensus for the optimal calorie intake of macronutrients in both diets [16]. According to several studies, fish consumption and the risk of developing T2DM is considered positive, inverse, or absent depending on geographical location and other factors that influence the type of fish consumed, preparation/confection methods, and contaminants (e.g., methylmercury) [16]. Nevertheless, the consumption of fish oil is recommended for a positive effect on lipoproteins and prevention of cardiac coronary diseases, being a subject of debate the discouragement the supplementation of ω-3 in diabetics [16]. Dairy consumption is recommended in T2DM prevention, especially fermented as yogurt, although the promotion of low-fat dairy products in this population is debatable [16]. Tropical and plant oils are also subjects of study in T2DM prevention and control, with evidence supporting the benefit of olive oil as part of the Mediterranean diet [16].

Regardless of the effects on body weight, physical inactivity is identified as an independent risk factor for T2DM that can be reduced by 20–60% with regular physical activity in a dose-response manner [8]. Glucose is transported by proteins called GLUT (Glucose Transporter), with GLUT4 being the predominant isoform in muscle, modulated by insulin and muscle contractions [18]. Insulin activates the intracellular transport of GLUT4 to the cell membrane through a complex signaling sequence, which is usually compromised in individuals with T2DM, but is stimulated during aerobic or anaerobic exercise [18]. Exercise allows for the increase of GLUT4 by muscle contractions and increased glucose uptake, which is corroborated with the normal values of glucose absorption in patients with T2DM submitted to exercise protocols [18]. Glycogen is the first source of energy for exercise through glycogenolysis, resorting to plasma glucose absorption and a release of free fatty acids when the first source of energy runs out [18]. In long-term activities, glycemic control is achieved by the use of intramuscular lipid reserves [18]. In healthy subjects during moderate/intense exercise, increased peripheral glucose uptake is accompanied by increased hepatic glucose output, allowing for the maintenance of plasma glucose values, except during prolonged exercise [18]. In subjects with T2DM, during moderate exercise, glucose uptake by muscles increases more strongly than their production, leading to a decline in plasma glucose levels [18]. However, the risk of hypoglycemia reduces in non-medicated individuals with insulin or their secretagogues, due to the decrease in plasma insulin levels, even in prolonged exercise [18]. The action of aerobic exercise on insulin effect varies with duration, intensity and subsequent diet, where a single session increases the action and glucose tolerance between 24 h and less than 72 h [18]. However, brief and intense aerobic exercise increases catecholamine plasma levels, causing an increase in glucose production, which can result in hyperglycemia for 1 h to 2 h since levels do not return to normal immediately after the activity ends [18].

## 3. Novel Therapeutic Targets for Naturally-Occurring Compounds 

### 3.1. Adiponectin

Adiponectin (*Acrp30*, *AdipoQ*, *GBP-28,* or *apM1*) is an endocrine factor, mainly secreted by the adipose tissue, but also by skeletal and cardiac myocytes and endothelial cells, with direct actions in the liver, skeletal muscle, and vasculature [19]. It exists in the circulation as varying molecular weight forms produced by multimerization, where the high-molecular weight complexes have apparent predominant action on metabolic tissues [19]. Adiponectin administration in human and rodents has insulin-sensitizing, anti-atherogenic, and anti-inflammatory effects with possible decreases of body weight [19]. In fact, low plasma adiponectin concentrations are associated with obesity and T2DM regardless of ethnic groups, where hypoadiponectinemia is more closely related to the degree of insulin resistance and hyperinsulinemia than the degree of adiposity and glucose intolerance [20]. As a result of the importance of adiponectin levels in the pathology, studies have been conducted aiming the discovery of new natural sources which promote adiponectin release. Some of these studies are reviewed by Ríos and colleagues, reporting different natural products for the treatment of T2DM from medicinal plants such as *Ipomoea batatas*, *Aronia melanocarpa,* and *Salacia reticulate*, as well as from mushroom *Agaricus blazei* [21]. 

### 3.2. Lipase Inhibition

Fat digestion involves gastrointestinal enzymes like pre-duodenal lipases (lingual and gastric lipases), pancreatic lipase, cholesterol-ester lipase, and bile-salt stimulated lipase [22]. Triglycerides are the most dietary fat ingested (90–95%). Their hydrolysis starts in the mouth, followed by acid stable gastric lipase in the stomach and synergistic action of gastric and colipase-dependent pancreatic lipase in duodenum [22]. As a result, monoglycerides and free fatty acids are formed, the latter being absorbed by enterocytes to synthetize new triglyceride molecules, which are transported by lipoproteins (chylomicrons) to different organs after a meal [22]. Pancreatic lipase is responsible for the hydrolysis of 50–70% of total dietary fats, highlighting it as the main lipid digesting enzyme [22,23]. 

#### 3.2.1. Pharmacological Approach

The inhibition of lipase leads to restored insulin production from β-cells protecting pancreas through decrease of lipid absorption. Orlistat is the most prescribed synthetic drug for the pathology, but with several side effects reported including steatorrhea, bloating, oily spotting, fecal urgency, fecal incontinence, and hepatic adverse effects [22,24,25]. Aditionally, the inhibition of fat absortion results in the need of vitamin supplementation because of the defficiency of fat-soluble vitamins in patients undergoing orlistat theraphy [22]. 

#### 3.2.2. Naturally-Occurring Lipase Inhibitors

Considering the numerous side effects related to the use synthetic lipase inhibitors, interest in the search for new natural inhibitors against pancreatic lipase is growing. Some of them are summarized in Table 1 and described below.

##### Plants

Roh & Jung reported inhibitory activity of porcine pancreatic lipase in crude extracts of *Rubi Fructus* (32.5%), *Corni Fructus* (34.8%), *Salicis Radicis et Cortex* (38%) and *Geranium nepalense* (31.4%) at 100 μg/mL [23]. 

5-methoxy-7-hydroxy-9,10-dihydro-1,4-phenanthrenequinone isolated from a methanol extract of the whole plant of *Dendobium formosum* at 50 μg/mL is related with non-competitive inhibition of alpha-glucosidase (96%) and pancreatic lipase (83%), reveling IC_50_ (half maximal inhibitory concentration) values of 126.88 ± 0.66 μM, and 69.45 ± 10.14 μM, respectively [24].

Hexane and methanol extracts of leaf essential oil of the Tunisian traditional medicine *Juniperus phonicea* also showed an inhibitory effect against alpha-amylase (IC_50_ = 30.15 μg/mL) and lipase (IC_50_ = 60.22 μg/mL), respectively, where the inhibitory capacity against the latter enzyme was coincident with their total phenolic compounds [25]. Ethanolic extracts of star apple *Chrysophyllum cainito* appears to present an inhibitory effect against pancreatic lipase (74.91%), and the concentration of its bioactive compounds in hexane showed 92.11% inhibition [26]. In fact, there are many studies involving the antipancreatic lipase properties of plants, including methanol extracts of fermented *Camellia japonica* (IC_50_ = 0.308 mg/mL) [27]; ethanol extracts of *Camelia sinensis* (IC_50_ = 0.5 mg/mL), *Ceratonia siliqua* (IC_50_ = 0.8 mg/mL), *Curcuma longa* (IC_50_ = 0.8 mg/mL), *Sarcopoterium spinosum* (IC_50_ = 1.2 mg/mL), and *Mentha spicata* (IC_50_ = 1.2 mg/mL) [28]; crude extract (IC_50_ = 0.84 mg/mL), ethyl acetate extract (IC_50_ = 0.88 mg/mL) and aqueous fraction (IC_50_ = 0.63 mg/mL) of *Oxalis cordata* [32]. *Eucalyptus globulus* and *Mentha viridis* where evaluated for their inhibitory potential against lipase from *Aspergillus niger* and extracted from olive mesocarp [33]. The investigators found that methanol extracts from *E. globulus* and *M. viridis* presented greatest inhibition against lipase of *Aspergillus niger* (78.92 and 72.75%, respectively) and against lipase of olive (82.65 and 75.75%, respectively), where phenolic compounds might be a major contributor for the lipases inhibition [33]. Black tea (*Camellia sinensis*) theaflavins coupled with thearubigins are characteristic polyphenols in black tea, resulting from the oxidation of tea leaves during fermentation process [29]. (−)-Epigallocatechin-3-gallate is the major polyphenol in green tea and inhibit lipase in a non-competitive way, with IC_50_ of 7.5 μmol/L [29]. Theaflavin-3,3’-digallate, theaflavin-3′-gallate, theaflavin-3-gallate and theaflavin inhibited pancreatic lipase with IC_50_ of 1.9; 4.2; 3 and >10 μmol/L, respectively, where the presence and location of the galloyl ester moiety were essential for inhibitory potency [29]. Oolong tea is a semifermented tea which presents inhibitory activity against lipase, because of its polyphenolic content, namely (−)-epigallocatechin-3-*O*-gallate (IC_50_ = 54.97 ± 1.32 μM), (−)-gallocatechin-3-*O*-gallate (IC_50_ = 54.68 ± 2.23 μM) and (−)-epicatechin-3-*O*-gallate (IC_50_ = 38.22 ± 0.89 μM) [30]. A methanol extract of *Hibiscus sabdariffa* (*Malvaceae*) also presented inhibitory effect against pancreatic lipase (IC_50_ = 35.8 ± 0.8 μg/mL) and alpha-amylase (IC_50_ = 29.3 ± 0.5 μg/mL), as well as the methanol extract of the leguminous *Tamarindus indica* (IC_50_ = 152 ± 7 μg/mL) against lipase and aqueous extract against alpha-amylase (IC_50_ = 139.4 ± 9 μg/mL) [34]. Commonly used Cameroonian spices where investigated for their in-vitro anti-amylase and anti-lipase activity [35]. This study revealed that aqueous extracts of *Aframomum danielli*, *Hypodapnis zenkeri, Echinops giganteus*, *Aframomum citratum*, *Xylopia aethiopica* had more than 75% inhibitory activity against pancreatic amylase, and *Xylopia aethiopica* (92.25%) and *Scorodophloeus zenkeri* (56.39%) were most effective against pancreatic lipase [35]. Masqsood and colleagues evaluate the inhibitory activity of different extraction conditions of *Lagenaria siceraria*, reveling that chloroform fractions showed maximal inhibitory effect with IC_50_ value of 157.59 μg/mL [36]. Additionally, the gas chromatography-mass spectrometry (GC/MS) analysis of the most active chloroform fractions showed the presence of hexadecanoic acid, methyl hexadecanoate, isopropryl palmitate, methyl 9,12-octadecadienate, and methyl 9,12,15-octadecatrienoate [36].

Oxalic and furoic acid, which are compounds derived from the oxidation of vitamin C to dehydroascorbic acid, were investigated for their inhibitory activity against pancreatic lipase [37]. In this study, furoic acid and oxalic acid revealed IC_50_ values of 2.12 ± 0.04 and 15.05 ± 0.78 mM, respectively, where the ultraviolet (UV) wavelength scanning and fluorescence quenching experiments proved that furoic acid inhibition was stronger than that of the other compound [37]. Both acids presented reversible inhibition with non-competitive and competitive action by furoic and oxalic acid, respectively [37]. Other compounds evaluated for their potential as lipase inhibitors include terpenes, like carnosic acid isolated from methanol extract of *Salvia officialis* at 36 μM (IC_50_ = 12 μg/mL) and bioactive-guided fractionation of this plant with isolation of carnosol, roylenoic acid,7-methoxyrosmanol, and triterpene oleanolic acid with IC_50_ values of 4.4; 35; 32; and 83 μg/mL, respectively [54]. Other terpenes include crocin from *Gardenia jasminoids* with an IC_50_ value of 28.63 μmol [54].

##### Polyphenolic Compounds—Fruits, Vegetables and Plants

The beneficial effect of polyphenols and their subgroups on lipid metabolism is extensively reported on literature. Flavonoids are the most common group of polyphenolic compounds in the human diet and are found ubiquitously in plants, mainly on leaves or shells, with the function of protecting them from harmful external influences [55]. Flavonoids can be classified, based on the degree of the oxidation of the C-ring, the hydroxylation pattern of the ring structure and the substitution of the C3-position, into: chalcones, dihydrochalcones, aurones, flavones, flavonols, dihydroflavonoles, flavanones, flavanols, anthocyanidins, leucoanthocyanidins, proanthocyanidins, bioflavonoids, and isoflavonoids [55]. On the other hand, phenolic acids occur as free fatty acids, esters, glycosides, or bound complexes, with antioxidant, anticarcinogenic, and antimicrobial activity, where hydroxycinnamic acids are phenolic compounds with major importance for secondary metabolism in plants occurring in fruits (apples, blueberries), vegetables (spinach, lettuce, potatoes), coffee, and cereals [55]. By-products rich in polyphenols from winemaking, were treated with pronase and viscozyme to improve the solubility of phenolics by Camargo and colleagues [48]. The study revealed that the inhibition of soluble phenolics against alpha-glucosidase and lipase increased from 75.6% ± 2.5% to 93.7 ± 0.5% and from 35.2 ± 0.2% to 45.5 ± 1.2%, respectively, in samples treated with pronase and from 84.5 ± 0.5% to 96.5 ± 2.9% and from 86.2 ± 0.3% to 94.3 ± 1.5%, respectively, with viscozyme [48]. Polyphenol-rich extracts of six common bean cultivars (*Phaseolus vulgaris*) showed inhibitory activity against alpha-amylase (IC_50_ values ranged from 69 ± 1.9 to 126 ± 3.2 μg/mL and from 107.01 ± 4.5 to 184.20 ± 5.7 μg/mL before and after cooking), alpha-glucosidase (IC_50_ values ranged from 39.3 ± 4.4 to 74.13 ± 6.9 μg/mL and from 51 ± 7.7 to 122.1 ± 5.2 μg/mL before and after cooking) and pancreatic lipase (IC_50_ values ranged from 63.11 ± 7.5 to 103.2 ± 5.9 μg/mL and from 92 ± 6.3 to 128.5 ± 7.4 μg/mL before and after cooking) [38]. Ethanol and methanol extracts from *Lovi* (Botoko plum) from *Flacourtia inermis*, found in Sri Lanka exhibited inhibitory activity against alpha-glucosidase (IC_50_ from 549 to 710 ppm), alpha-amylase (IC_50_ from 1021 to 1949 ppm) and lipase (IC_50_ from 1290 to 2096 ppm), where (S)-malic acid was characterized as the active principle for this inhibition effect [40]. Additionally, this fruit has great polyphenol (1.28 g gallic acid equivalents per 100 g of fresh fruit) and anthocyanin (108 mg cyaniding-3-glucoside equivalents per 100 g fresh fruit) content [40]. Acacia polyphenol extracted from the bark of the black wattle tree (*Acacia mearnsii*) was tested for its in-vitro inhibitory activity against lipase and glucosidase, as well as its effects on absorption of orally administered olive oil, glucose, sucrose, and starch solution in ICR mice [41]. The results showed that acacia polyphenol inhibited the activity of lipase (IC_50_ = 0.95 mg/mL), maltase (IC_50_ = 0.22 mg/mL) and sucrase (IC_50_ = 0.60 mg/mL) and inhibited the rise plasma triglyceride concentration after olive oil loading, rise in plasma glucose concentration after maltose (more potent), glucose and sucrose loading [41]. Water extract of *Juglans mandshurica* strongly inhibited pancreatic lipase in-vitro (IC_50_ = 2.3 mg/mL for 50% of inhibition) with further evaluation of this activity in isolated compounds reveling potent inhibitory effect of 1,4,8-trihydroxynaphthalene-1-O-β-D [6 ’-O-(3′’,4′’,5′’-trihydroxybenzoyl)] glucopyranoside (88% at 1 mM) [42]. Simão and colleagues tested the inhibitory potential of an aqueous extract from the leaves of three cultivars of *Psidium guajava* (guava) on α-amylase, α-glucosidase, lipase and trypsin enzymes. These studies were done in the presence or absence of simulated gastric fluid and the content of phenolic compounds was determined [43]. The study revealed that all cultivars showed the same phenolic composition but in different proportions, where catechin was the major compound in Paluma cultivar, epigallocatechin gallate, and revesterol in Pedro Sato and syringic acid, *o*-coumaric acid, and quercetin for Século XXI [43]. The enzyme inhibition occured in major proportion for Século XXI cultivar against alpha-amylase before the exposure to gastric fluid (14410.60 ± 38 inhibited enzyme unit in μmol·min^−1^·g^−1^) and Paluma against alpha-glucosidase before (28.82 ± 0.02 inhibited enzyme unit in μmol·min^−1^·g^−1^) and after exposure (2.59 ± 0.06 inhibited enzyme unit in μmol·min^−1^·g^−1^) [43]. Pedro Sato revealed the highest inhibitory effect against lipase before (36.45 ± 0.68 inhibited enzyme unit in μmol·min^−1^·g^−1^) and after exposure (43.33 ± 1.80 inhibited enzyme unit in μmol·min^−1^·g^−1^) [43]. The three cultivars presented an inhibitory effect against trypsin activity before exposure, with significant reduction after exposure ranging from 84.88% for Paluma and 91.02% for Pedro Sato [43]. The investigators concluded that the inhibition of digestive enzymes could probably be explained by the presence of phenolic compounds in the cultivars aqueous extracts [43].

Saponins are composed by sugars attached to a steroid or triterpene, which are primary constituents of the roots and rhizomes of various plants, being responsible for a diversity of biological effects [54]. These plants with potential against lipase include: *Platycodin grandiflorum* (isolated platycodin D from radix), *Scabiosa tschiliensis* (prosapogenin 1b at 0.12 mg/mL), *Accanthopanax sessiloside* (sessiloside and chiisanoside IC_50_ values of 0.36 and 0.75 mg/mL, respectively), *Panax japonicus*, *Dioscorea nipponica* (dioscin IC_50_ of 20 μg/mL), and *Cyclocarea paliurus* (extract present IC_50_ of 9.1 mg/mL) [54]. Tea saponin, oleanolic acid, betulin, ginsenoside Ro, and ginsenoside Rd were investigated in-vitro through comprehensive evaluation of pancreatic lipase activity [31]. Tea saponin inhibit lipase in a competitive way, while the other triterpenoid saponins act non-competitively, where the inhibitory efficiency towards lipase was different in each saponin possibly due to the different structures of this compounds [31]. The EC_50_ value was 0.885 mM for Ro; 0.895 mM oleanolic acid; 0.958 mM for botulin; 242 mM for tea saponin; and 388 mM for Rd [31]. In another study with plant species of Madeira *Argyranthemum pinnatifidum* leaves and flowers exhibit inhibition activity against yeast alpha-glucosidase (IC_50_ = 0.57 ± 0.03 mg/mL and 0.81 ± 0.02 mg/mL, respectively) and alpha-amylase (IC_50_ = 1.55 ± 0.04 mg/mL and 2.18 ± 0.05 mg/mL, respectively) [44]. Alpha-amylase was also inhibited by *Artemisia argentea* leaves and flowers (IC_50_ = 1.81 ± 0.03 mg/mL and 2.48 ± 0.04 mg/mL, respectively), *Helichrysum devium* (IC_50_ = 1.85 ± 0.06 mg/mL and 2.39 ± 0.03 mg/mL, respectively), and *Helichrysum melaleucum* (IC_50_ = 1.71 ± 0.02 mg/mL and 2.15 ± 0.05 mg/mL, respectively) [44]. Lipase was inhibited by *Phagnalon lowei* leaves (IC_50_ = 3.05 ± 0.07 mg/mL) [44]. Pits from Tunisian date palm variety Kentichi, exhibit inhibitory activity against α-amylase and lipase with ethyl acetate (IC_50_ = 25.4 ± 0.6 μg/mL and IC_50_ = 5.4 ± 0.6 μg/mL, respectively) and methanol (IC_50_ = 0.072 ± 0.003 μg/mL and IC_50_ = 1.21 ± 0.23 μg/mL, respectively) extracts [45]. Other examples of plants with an inhibitory effect against lipase include 3-*O*-trans-p-coumaroyl actinidic acid isolated from roots of *Actinidia arguta* (IC_50_ = 14.95 μM) [46]; and baicalin, wogonin, and oroxylin A flavonoids from *Scutellaria baicalensis* (IC_50_ of 229.22 ± 12.67, 153.71 ± 9.21 and 56.07 ± 4.90 μM, respectively) [47].

Condensed tannins called proanthocyanidins are found in the insoluble fraction of plant-derived foodstuffs (e.g., grape seed and skin, red wine, apples), where type-B are the more abundant and type-A are abundant in vegetables like peanut skin, avocados, and cranberries [49]. When the degree of polymerization of proanthocyanidins is >3, they are poorly absorbed in the intestine, reaching the colon as substrate for specific bacteria of the resident microbiota, contributing to the modulation of the composition of the colonic microbiota [49]. In the lumen of the small intestine, high molecular-weight condensed tannins may interfere with macronutrients, bile salts, mucosal alpha-glucosidase, and pancreatic enzymes (alpha-amylase, lipase and proteases) decreasing nutrient digestibility, being frequently considered as antinutrients [49]. In fact, grape extract exhibits inhibitory activity against lipase with an IC_50_ value of 8.6 ± 1.1 mg/mL [49].

##### Polyphenolic Compounds—Seaweed

Seaweed are extensively studied for their health benefits; namely, inhibition of certain crucial enzymes for the control/prevention of DM. These studies include: ethanol extract of dried *Kappaphycus striatus* with 92% inhibition against lipase activity and 88% for fresh *Euchema denticulatum* against alpha-amylase [50]; purified active inhibitor caulerpenyne from ethyl acetate extraction of *Caulerpa taxifolia* competitively inhibited 50% of lipase activity using emulsified triolein and dispersed 4-methylumbelliferyl oleate as substrates at 2 mM and 13 μM, respectively [51]. Additionally, methanolic extract of brown seaweed *Eisenia bicyclis* revealed an inhibitory effect of pancreatic lipase, with IC_50_ values ranging from 37.2 ± 2.3 to 12.7 ± 1 μM for fucofuroeckol A and 7-phloroeckol, respectively [52]. Brown seaweed *Saccharina japonica* fermented by red mold *Monascus purpureus* inhibited alpha-amylase (IC_50_ = 0.98 ± 0.10 mg/mL), rat intestinal alpha-glucosidase (maltose IC_50_ = 0.02 ± 0.07 mg/mL and sucrose IC_50_ = 0.08 ± 0.13 mg/mL) and lipase (IC_50_ = 4.98 ± 0.85 μg/mL) [53]. Austin and colleagues, verified that a polyphenol-rich extract from the edible seaweed *Ascophyllum nodosum* inhibited pancreatic lipase activity in an oil-based turbidimetric assay (IC_50_ = 200 μg gallic acid equivalents per assay) as well as its purified phlorotannin-enriched fraction (IC_50_ = 60 μg gallic acid equivalents per assay) [39].

##### Alginates from Algae

Alginates are dietary fibers found in the cell walls of brown seaweed and certain bacteria and comprised of mannuronic and guluronic acid. Wilcox and colleagues found that alginate inhibited pancreatic lipase by a maximum of 72.2 ± 4.1% with synthetic substrate (DGGR) and 58 ± 9.7% with olive oil as substrate, where High-G alginates from *Laminaria hyperborea* seaweed has shown the most potent inhibitory activity than High-M alginates from *Lessonia nigrescens*, revealing that the alginate structure is related to the inhibition [56].

##### Metabolic Products from Microorganisms

According to a review by Birari and Bhutani, metabolic products from microorganisms can also inhibit pancreatic lipase [54]. These products include: lipstatin isolated from *Streptomyces toxytricini* (IC_50_ = 0.14 μm); panclicins from *Streptomyces* sp. (panclicins A, B, C, D, E with IC_50_ values of 2.9; 2.6; 0.62; 0.66; 0.89 μM, respectively); valilactone from *Streptomyces albolongus* (IC_50_ = 0.14 nm); ebelactones A and B from *Streptomyces aburaviensis* (IC_50_ of 3 and 0.8 nm/mL, respectively); esterastin from *Streptomyces lavendulae* (IC_50_ = 0.2 ng/mL); caulerpenyne from *Caulerpa taxifolia* (IC_50_ of 2 mM and 13 μM with emulsified triolein and dispersed 4-methylum-belliferyl oleate as substrates, respectively); vibralactone from *Boreostereum virans* (IC_50_ = 0.14 μg/mL); and percyquinnin from *Basidiomycete stereum complicatum* (IC_50_ = 2 μm) [54].

### 3.3. PTP1B Inhibition

The enzyme responsible for the reversal insulin receptor auto phosphorylation is a tyrosine phosphatase known as PTP1B (protein tyrosine phosphatase 1B), where inhibition results in a prolonged insulin signaling cascade, increasing insulin sensitivity [57]. However, low selectivity over the other protein tyrosine phosphatases, ubiquitously expressed, and poor cell permeability are two major challenges in the discovery of efficient inhibitors [58]. Nevertheless, there are potential PTP1B inhibitors from natural sources, some of these being described in Table 2.

Polyphenols mulberrofurans C, J and F from *Morus alba* showed the best inhibitory effect against PTP1B in a study by Huang and colleagues, with IC_50_ values of 0.72 ± 0.09, 0.60 ± 0.07, and 0.57 ± 0.16 μM, respectively [59]. Caffeoylquinic acid derivate chlorogenic acid from leaves of *Artemisia princeps* is a noncompetitive inhibitor with IC_50_ value of 11.1 μM using ursolic acid (known PTPB1 inhibitor) as positive control (IC_50_ = 3.1 μM) [60]. Isoderrone, derrone, alpinumisoflavone, and mucusisoflavone isolated from ethanol-water extract of *Ficus racemosa* exhibit inhibitory activity against PTP1B with IC_50_ values of 22.7 ± 1.7, 12.6 ± 1.6, 21.2 ± 3.8, and 2.5 ± 0.2 μM, respectively [61]. Ezzat and colleagues reviewed marine-derived bioactive molecules as PTP1B inhibitors where they mentioned this activity in sulfircin (sesterpene sulfate) isolated from deep-water sponge *Ircinia*, bromophenols from red algae *Rhodomela confervoides* (IC_50_ from 0.8 to 4.5 μM), two bromophenols from Indonesian marine sponge *Lamellodysidea herbacea* (IC_50_ 0.9 and 1.7 μM), and others from red algae *Symphyocladia latiuscula* (IC_50_ 3.9, 4.3 and 2.7 μM) [62]. Other compounds mentioned in this review include brominated metabolites from red algae *Laurencia similis*, polybromodiphenyl ether derivatives from marine sponge *Lamellodysidea herbacea*, eckol, and its derivatives isolated from brown algae *Ecklonia stolonifera* and *Eisenia bicyclis* [62].

### 3.4. DPP4 Inhibition

#### 3.4.1. Incretins and DPP4

Incretins are hormones produced by intestinal enteroendocrine cells on ingestion of glucose [2,11]. Incretins such as GIP (glucose-dependent insulinotropic polypeptide) and mainly GLP-1 (glucagon-like peptide-1) are responsible for the incretin effect, resulting from the observation that oral glucose is more effective in promoting insulin secretion than intravenous glucose [14]. Thus, 70% of postprandial insulin is secreted by pancreatic β-cells as a response to this incretin effect, being both this effect and physiological activity of the incretins reduced in T2DM [2,11,63]. GLP-1, in addition to insulin-tropic action in response to high concentrations of glucose, allows weight loss through the mediation of satiety and reduction of gastric emptying rate, being also responsible for the suppression of glucagon secretion by pancreatic α-cells in a glucose- dependent process, allowing the cessation of hepatic glucose secretion [14,63]. When plasma glucose levels return to normal, the inhibitory effect under alpha-cells ceases, preventing hypoglycemia [63]. GIP, like GLP-1, allows for the increase of the insulin secretion and inhibition of the glucagon discharge, emphasizing this by stimulating the secretion of glucagon during hypoglycemia [63]. GLP-1 and GIP are rapidly degraded by the enzyme dipeptidyl peptidase-4 (DPP4) widely distributed throughout the body [64].

#### 3.4.2. Pharmacological Approach

Active GLP-1 has a half-life of 1–2 min and its inhibition constitute an efficient pharmacological approach for T2DM treatment [4]. In fact, a new class of drugs has emerged, which allows the stimulation of endogenous insulin secretion, preventing the rapid degradation of incretin hormones, through the inhibition of DPP4 [65]. DPP4 inhibitors, or gliptins, are effective as monotherapy and in combination therapy, allowing the reduction of HbA1c without causing hypoglycemia or weight gain [11]. Gliptins can be classified as peptidomimetics (designed to mimic the N-terminal dipeptide that is cleaved by DPP4) such as Vildagliptin and Saxagliptin, or non-peptidomimetics such as Linagliptin and Sitagliptin [66]. The most widely antihyperglycemic agent prescribed worldwide is metformin, despite its association with vitamin B_12_ deficiency and contraindication in patients with chronic kidney disease [4,5]. Another class of drugs based on the incretin effect refers to GLP-1 analogues, namely exenatide, which demonstrates statistically significant body weight reduction when compared to insulin/placebo [14]. Besides the existing pharmacological strategies, side effects and contraindications reveal the need for new natural therapies for inhibition for DPP4 [67]. In fact, the prolonged usage of these medications causes side effects such as pancreatitis, angioedema, infective disorders, pancreatic cancer, thyroid cancer, and severe joint pain [68], increasing interest of the scientific community for the development of natural inhibitors (Table 3).

#### 3.4.3. Naturally-Occurring DPP4 Inhibitors

##### Peptides

Dietary proteins have been increasingly recognized as precursors of a variety of bioactive peptides, improving various aspects of human health [74]. These bioactive peptides are present in inactive forms in food, being activated once released from the proteins by enzymatic or acid hydrolysis, microbial fermentation or processing methods, and their biological activity is determined by their native amino acid composition and sequence [74]. In-silico analyses are useful to determine the frequency of the occurrence of bioactive peptides within a dietary protein (simulation of protein hydrolysis by bioinformatic tools to calculate a number of bioactive peptides found in a given dietary protein) and binding modes by docking analysis (simulate the binding and interactions between peptides and enzymes, like DPP4, in order to evaluate the inhibitory effects of the peptides) [74]. Peptides can inhibit DPP4 with competitive (Xaa-Pro, Pro-Xaa, Xaa-Ala, and food derived peptides with proline at their P_1_ position), non-competitive and uncompetitive (N-terminal with tryptophan amino acid) and mixed-type modes of action, exerting their effect by binding either at the active site and/or outside the catalytic center of the enzyme [75]. The DPP4 inhibitory activity of bioactive peptides has been associated with some structural characteristics like length, isoelectric point, hydrophobicity, and net charge of the peptides, being the most predominant factor the specific amino acid sequence [75]. Other characteristics associated with potent inhibitory effect are: branched-chain amino acid or an aromatic residue with a polar group in the side-chain (tryptophan) at their N-terminal and/or a proline residue, were C-terminal amino acid also influences its potency since both are involved in the interaction with DPP4 [75].

DPP4 is known to act on substrates with proline or other small-uncharged residues such as serine and alanine at their penultimate amino acid position [75]. Because of high content in proline residues, collagen from fish and mammals has also attracted notable attention as a potential source of DPP4 inhibitory peptides [66,75]. To date, protein hydrolysates and bioactive peptides from cow’s milk have been the most extensively investigated sources of DPP4 inhibitors. In fact, according to a study with in-silico approach, caseins from cow’s milk (beta-casein with an occurrence frequency value of 0.249) and collagens from bovine meat and salmon (occurrence frequency values of 0.380 and 0.305, respectively) appeared to be the richest potential sources of DPP4 inhibitors, where Gly-Ala, Gly-Pro and Pro-Gly were the most frequently occurring sequences [69]. According to a review, peptides from Atlantic salmon skin gelatin (Gly-Pro-Gly-Ala and Gly-Pro-Ala-Glu sequence), tuna cooking juice (Pro-Gly-Val-Gly-Gly-Pro-Leu-Gly-Pro-Ile-Gly-Pro-Cys-Tyr and Cys-Ala-Tyr-Gln-Trp-Gln-Arg-Pro-Val-Asp-Arg-Ile-Arg and Pro-Ala-Cys-Gly-Gly-Phe-Tyr-Ile-Ser-Gly-Arg-Pro-Gly), Japanese rice bran, Native American amaranth, and Gouda cheese also present the DPP4 inhibitory effect [66].

Peptides which were not isolated from hydrolysates but are likely to occur in the sequence of dietary proteins, were synthetically produced and studied for their effect on DPP4 activity [75]. Diprotin A (tripeptide IPI) is the most potent peptide with DPP4 inhibitory activity (IC_50_ ≈ 4 μM) and can be found in the sequence of k-casein. However, not as effective as diprotin A, the peptides WR, IPIQY, and WCKDDQNPHS found in the sequence of lactoferrin, k-casein, and α-lactalbumin, respectively, are among the most potent food protein-derived DPP4 inhibitors reported to date [75]. However, these peptides could not be released from dietary proteins during digestion or enzymatic proteases, like those isolated and identified in hydrolysates and thus unavailable for inhibitory effect of DPP4 [75]. Nongonierma and colleagues tested a selection of synthetic dipeptides and milk protein hydrolysates for their DPP4 inhibitory properties, and their superoxide and 2,2-diphenyl-1-picrylhydrazyl (DDPH) radical scavenging activities [70]. The study revealed superoxide and DPPH scavenging activity and the DDP4 inhibitory effect, by dipeptide Trp-Val (EC_50_ 39.75 ± 0.01 mg/mL; EC_50_ 0.07 ± 0.01 mg/mL; IC_50_ 0.020 ± 0.001 mg/mL respectively) and lactoferrin hydrolysate LFH1 (EC_50_ 0.10 ± 0.01 mg/mL; EC_50_ 1.15 ± 0.40 mg/mL; IC_50_ 1.088 ± 0.106 mg/mL). However, the dipeptide Ala-Leu had bigger superoxide scavenging activity (EC_50_ 1.74 ± 0.01 mg/mL) than Trp-Val, which was the dipeptide with greater DPPH scavenging activity [70]. Besides LHF1, casein hydrolysate (CasH2) also showed a potent inhibitory effect on DPP4 (IC_50_ 0.882 ± 0.057 mg/mL) [70].

##### Polyphenolic Compounds: Fruits, Vegetables, and Plants

The potential of phenolic compounds in the treatment and prevention of obesity is due to their thermogenic effects, which corresponds to the ability to oxidize fat and decrease intestinal absorption of fats and carbohydrates, resulting in the inhibition of digestive enzymes with consequent weight loss [43]. Beneficial health effects of fruits and vegetables in the diet have been attributed to their high phenolic content, such as flavonoids. With the purpose of inhibit DPP4 and PTPB1, Bower and coworkers studied the ability of greenhouse-grown and commercially purchased Greek oregano (*Origanum vulgare*), marjoram (*Origanum majorana*), rosemary (*Rosmarinus officinalis*), and Mexican oregano (*Lippia graveolens*) [57]. Greenhouse herbs were richer in polyphenols than the commercial ones. Mexican oregano and marjoram were the best inhibitors of PTP1B (32.4–40.9% at 500 μM) with cirsimaritin, naringenin, hispidulin, eriodictyol, and carnosol in their composition according to LC-ESI-MS method [57]. According to computational modeling, the last three phytochemicals have the best binding affinities for DPP4, but biochemically the best inhibitors of DPP4 were cirsimaritin (IC_50_ = 0.43 ± 0.07 μM), hispidulin (IC_50_ = 0.49 ± 0.06 μM) and naringenin (IC_50_ = 2.50 ± 0.29 μM), found in rosemary and Mexican oregano extracts [57]. Fan and colleagues investigated the DPP4 inhibitory effect of well-characterized anthocyanins isolated from berry wine blends, and twenty-seven other phenolic compounds commonly found in citrus, berry, grape and soybean using luminescence assay and computational modeling (for the most potent compounds) [71]. Malvidin-3-galactoside and cyaniding-3-glucoside were the main anthocyanins present in blueberry wine, while delphinidin-3-arabinoside was predominant in the blackberry wine [71]. Anthocyanins from blueberry-blackberry wine blends (IC_50_ = 0.07 ± 0.02 to > 300 μM) and phenolics resveratrol (IC_50_ = 0.6 ± 0.4 nM), luteolin (IC_50_ = 0.12 ± 0.01 μM), apigenin (IC_50_ = 0.14 ± 0.02 μM) and flavone (IC_50_ = 0.17 ± 0.01 μM) exhibit the most strongly inhibiting activity, where phenolics present IC_50_ values lower than diprotin A (IC_50_ = 4.21 ± 2.01 μM) [71]. According to computational modeling, resveratrol and flavone were competitive inhibitors and luteolin and apigenin docked in a noncompetitive manner [71].

##### Polyphenolic Compounds: Seaweed

Seaweed (also called algae) are simple unicellular (microalgae) or multicellular organisms (macroalgae), with rudimentary conductive tissues, presenting a high range of morphological and reproductive level variation that allows their division into different phyla and classes [76]. The presence of chlorophyll, production of the same carbohydrates, proteins and metabolic pathways, render algae biochemically similar to plants, differing in the absence of embryo and multicellular envelope around sporangia and gametangia in algae (except freshwather gren algae, charophytes) [76]. Marine macroalgae, are rich in bioactive compounds in the form of polyphenols, carotenoids, vitamins, phycobilins, phycocyanins, and polysaccharides, known for their benefits to human health [1,76]. An in-vitro assay revealed that ethanolic precipitates of *Sargassum binderi*, *Padina sulcata,* and *Turbinaria conoides* had inhibitory activity against DPP4 (IC_50_ = 2.194; 2.306 and 3.594 mg/mL, respectively) [64]. Additionally, the same study evaluated the viability of pGIP/neo STC-1 cells by measurement of cell membrane integrity by means of the Tryptan blue exclusion assay [64]. The evaluation revealed that water extracts of *S. binderi*, *P. sulcata,* and *T. conoides* allowed for the stimulation of GIP secretion of 5.46; 4.92 and 5 pM GIP per million cells per hour at 2.5; 10; 2.5 mg/mL, respectively [64]. Furthermore, the butanol fraction of *S. binderi* and *P. sulcata* allows for the stimulation of GIP secretion of 56.38 and 40.67 pM, respectively, GIP per million cells per hour at 5 mg/mL [64]. Another study revealed that methanol extract of brown seaweeds *Sargassum wightii* and *Sargassum polycystum* has an inhibitory effect against DPP4 (IC_50_ = 38.27 μg/mL and IC_50_ = 36.94 μg/mL, respectively) and acetone extract with moderate antioxidant activity (43% and 22%) at a concentration of 1000 μg/mL according to DPPH free radical scavenging activity method [65]. The same group of investigators found that methanol extract of brown seaweed *Turbinaria conoides* possesses inhibitory activity against DPP4 (55.4%, IC_50_ = 55.2 μg/mL) at a concentration of 80 μg/mL and significant scavenging ability on DPPH (65%) at a concentration of 1000 μg/mL of acetone extract [72].

Mangiferin is a glucosyl xanthone and is the major phytochemical in *Mangifera indica* (family of *Anacardiaceae*). It has strong antioxidant, antilipid peroxidation, immunomodulation, antidiabetic cardiotonic, hypotensive, wound healing, antihyperlipidemic, antiatherogenic, and antidegenerative properties [68]. Suman and colleagues studied the influence of mangiferin and synthetic drugs (metformin and vildagliptin) using two control groups of adult Wistar rats (normal control and diabetic control feed for 10 weeks with distilled water and a high-fat diet, respectively) [68]. The other subjects where fed for 10 weeks with a high-fat diet and subsequent streptozotocin (STZ)-induced T2DM (40 mg/kg) after 3 weeks, followed by the administration of metformin, vildagliptin or mangiferin from the fifth week to tenth week daily [68]. The study revealed a high DPP4 inhibitory effect of mangiferin (89 ± 8%) when compared with synthetic drugs (90 ± 7% for VIL and 84 ± 8% for sitagliptin) according to ELISA kit (Enzyme-Linked Immunosorbent Assay) [68]. Mangiferin permitted the reduction in blood glucose, HbA1c levels and MDA levels (marker of lipid peroxidation in organs like liver, heart and kidney) [68]. Additionally, this xanthone improves insulin sensitivity and C-peptide levels, showing a favorable effect on inflammatory markers hs-CRP [68]. Total cholesterol (*p* < 0.001), triglycerides (*p* < 0.001), LDL (*p* < 0.01), and atherogenic index (*p* < 0.01) were significantly reduced and HDL was increased (*p* < 0.01) in mangiferin and standard drugs treated groups [68]. Other marine sources with health benefits have been studied, such as sponges and anemones, where aqueous extracts of sponge *Xetospongia muta* and sea anemones *Bunodosoma granulifera* and *Bartholomea annulata* present inhibitory activity against DPP4 (0.82; 2.26 and 1.78 U/mg, respectively) [73].

### 3.5. Alpha-Amylase and/or Alpha-Glucosidase Inhibition

Pancreatic alpha-amylase allows the hydrolysis of carbohydrates through the breakdown of α-1,4-glycosidic bonds, forming linear and branched oligosaccharides, which are subsequently converted to glucose [12,13,65]. Such a conversion is catalyzed by the intestinal alpha-glucosidase, allowing its absorption into the bloodstream [12,13]. The inhibition of both enzymes, will allow to reduce the postprandial hyperglycemia by delayed digestion of carbohydrates and intestinal absorption of glucose [12,13,77].

#### 3.5.1. Pharmacological Approach

Acarbose, miglitol and voglibose are examples of inhibitors, where the former shows an excessive inhibitory activity of pancreatic alpha-amylase with consequent abnormal bacterial fermentation of carbohydrates in the colon, resulting in side effects such as flatulence, bloating, and possible diarrhea [77]. As a result of several side effects and high costs of pharmacological control of pathology, the scientific community is constantly in the search for natural sources with inhibitory effect against alpha-amylase and alpha-glucosidase, being summarized in Table 4 and Table 5, respectively, and described below.

#### 3.5.2. Naturally-Occurring Alpha-Amylase and/or Alpha-Glucosidase Inhibitors

##### Peptides

Protein hydrolysate from Chinese giant salamander (*Andrias davidianus*) was evaluated for its potential inhibitory activity against alpha-amylase and alpha-glucosidase, with further purification and identification of antidiabetic peptides [78]. The peptides amino acid sequences were Cys-Ser-Ser-Val, Tyr-Ser-Phe-Arg, Ser-Ala-Ala-Pro, Pro-Gly-Gly-Pro, and Leu-Gly-Gly-Gly-Asn with alpha-amylase IC_50_ values of 13.76 × 10^3^, 10.82 × 10^3^, 4.46 × 10^3^, 4.23 × 10^3^ and 2.86 × 10^3^, respectively; and with alpha-glucosidase IC_50_ values of 206.00, 162.00, 66.90, 63.50, and 42.93 μg/mL, respectively [78]. Garza and colleagues also reported the inhibition of alpha-amylase by valoneaic acid dilactone obtained from banaba (*Lagerstroemia speciosa*), ethanol extract of chestnut astringent skin, and a purified compound isolated from white beans (*Phaseolus vulgaris*) [22].

##### Polyphenolic Compounds: Fruits, Vegetables, and Plants

Extracts from grape seed, green tea, and white tea were evaluated for their potential to inhibit alpha-amylase and alpha-glucosidase [79]. Grape seed extract exhibited the best inhibitory effect against alpha-amylase (IC_50_ = 8.7 ± 0.8 μg/mL), followed by green tea extract (IC_50_ = 34.9 ± 0.9 μg/mL), and white tea (IC_50_ = 378 ± 134 μg/mL) [79]. However, green tea extract revealed the best inhibitory effect on alpha-amylase (IC_50_ = 0.5 ± 0.1 μg/mL), followed by grape seed extract (IC_50_ = 1.2 ± 0.2 μg/mL) and white tea extract (IC_50_ = 2.5 ± 0.4 μg/mL) [79]. Another study evaluated the alpha-amylase and alpha-glucosidase inhibitory effect of acylated favonol tetraglycoside (camellikaempferoside) and 14 other flavone glycosides isolated from *Camelia sinensis* [80]. The investigators found that kaempferol monoglycoside showed inhibitory activity against alpha-glucosidase (IC_50_ = 40.02 ± 4.61 μM) and kaempferol diglycoside against alpha-amylase (IC_50_ = 0.09 ± 0.02 μM) [80]. Aqueous extracts of *Morinda lucida* present high inhibitory effect against alpha-amylase with IC_50_ = 2.30 mg/mL and α-glucosidase IC_50_ = 2.00 mg/mL through a competitively and mixed noncompetitive mode of inhibition, respectively [81]. Besides these plant extracts, there are others that revealed an inhibitory activity against alpha-amylase and alpha-glucosidase, such as: *Senna surattensis* ethanolic extract (IC_50_ = 123.95 μg/mL for α-amylase) [82], leaf acetone extracts of *Picralima nitida* (IC_50_ = 6.50 mg/mL for α-amylase and IC_50_ = 3.00 μg/mL for α-glucosidase) [83], hydroxytyrosol derivate from the phenolic compound oleuropein present in *Olea europea* (75%, IC_50_ = 150 μM at 600 μM for α-amylase) [84] and ethanol-free extract of *Abutilon indicum* (IC_50_ = 191.64 mcg/mL for α-amylase and IC_50_ = 207.13 mcg/mL for α-glucosidase) [85]. Aqueous methanol and n-butanol extracts of air-dried aerial parts of *Ononis angustissima* flowers, leaves and stems reveled strong inhibitory activity against alpha-glucosidase (IC_50_ = 0.94 mg/mL and IC_50_ = 0.99 mg/mL, respectively) [86]. However, the stronger inhibitory effect against alpha-amylase was achieved with aqueous methanol and distilled water extracts (IC_50_ = 2.01 mg/mL and IC_50_ = 2.52 mg/mL, respectively) [86]. Alpha-amylase and alpha-glicosidase are inhibited by the root ethanolic extract of *Cissus cornifolia*, with IC_50_ value of 22.75 ± 1.23 μg/mL and 2.81 ± 0.97 μg/mL, respectively, where aqueous root extract revealed IC_50_ value of 33.70 ± 3.75 μg/mL and 37.48 ± 2.35 μg/mL, respectively [87]. Ethanolic extracts (70% ethanol) of medicinal plant roselle (*Hibiscus sabdariffa*) has potent inhibitory effect against alpha-amylase and alpha-glicosidase (47.34 and 73.08%, respectively) with IC_50_ values of 41.77 μg/mL for the former and 18.09 μg/mL for the latter [88]. Different commercially available chili peppers in Thailand where evaluated for their inhibitory activity against alpha-amylase and alpha-glicosidase [89]. “Sweet Pepper” and “Green Chinda” aqueous ethanolic extract (70% *v*/*v*) at 5 mg/mL exhibit the highest inhibitory activity against alpha-glicosidase (66%) and alpha-amylase (58%) [89]. On the other hand, a study revealed that purple flesh tubers extract has potent inhibitory activity against alpha-amylase, alpha-glicosidase, and aldose redutase (addressed ahead) with IC_50_ values of 25, 42 and 32 μg/mL respectively [90]. This study corroborates the opinion of some nutritionists that purple and red potato cultivars are a healthier choice for diabetic patients, rather than other types of potatoes, owing to their high levels of polyphenolic compounds with potent antioxidant activity [90].

##### Seaweed

Dieckol and eckol from brown algae *Eisenia bicyclis* inhibit alpha-amylase [91], while bromophenols 2,4,6-tribromophenol and 2,4-dibromophenol, from purified red algae *Grateloupia elliptica* shown inhition against *Saccharomyces cerevisiae* alpha-glucosidase (IC_50_ = 60.3 and 110.4 μM, respectively) and *Baccilus stearothermophilus* alpha-glucosidase (IC_50_ = 130.3 and 230.3 μM, respectively) [92]. Furthermore, diphlorethohydroxycarmalol (DPHC), a kind of phlorotanin, isolated from brow algae *Ishige okamure*, evidenced an inhibitory effect against alpha-glucosidase (IC_50_ = 0.16 mM) and alpha-amylase (IC_50_ = 0.53 mM) without cytotoxic effect in human umbilical vein endothelial cells in concentrations from 0.49 to 3.91 mM [93]. The inhibition of alpha-amylase also occurs in methanol (72%), ethanol (65%) and ethyl acetate (70%) extracts of green algae *Cladophora rupestris* and alpha-glucosidase in methanol (67%) and ethyl acetate (61%) extracts both inhibitions at 1000 μg/mL [94]. Phenolic extracts from red algae *Palmaria sp*. and brown algae *Alaria sp*. and *Ascophyllum sp.* (complete inhibition at 2–5 μg/mL, IC_50_ ≈ 0.1 μg/mL) show inhibitory effect against alpha-amylase, where the last also inhibits alpha-glucosidase (IC_50_ ≈ 19 μg/mL) [95]. Other seaweed with inhibitory activity against alpha-amylase and alpha-glucosidase include: *Ulva reticulata* and *Garcilaria edulis* [96]. In this point of view, marine resources are a valuable resource of powerful bioactive compounds for natural therapeutic strategies, especially for T2DM.

**Table 4:** Natural alpha-amylase inhibitors.

Compound	Source	Method	Results	Reference
Diphlorethohydroxycarmalol (DPHC)	*Ishige okamure*	In vitro	IC_50_ 0.53 mM	[93]
-	*Cladophora rupestris*		Methanol (72%), ethanol (65%) and ethyl acetate (70%) extracts	[94]
-	*Palmaria sp*.		Complete inhibition at 2–5 μg/mL, IC_50_ ≈ 0.1 μg/mL	[95]
	*Alaria sp*.			
	*Ascophyllum sp*			
-	Aqueous extract of *Ulva reticulata*		89.1 ± 0.96% inhibition after 8 h of extraction	[96]
-	Aqueous extract of *Gracilaria edulis*		87.86 ± 3.4% inhibition after 8 h of extraction	
-	Grape seed extract		IC_50_ 8.7 ± 0.8 μg/mL	[79]
-	Green tea extract		IC_50_ 34.9 ± 0.9 μg/mL	
-	White tea extract		IC_50_ 378 ± 134 μg/mL	
Kaempferol diglycoside (Figure 1k)	*Camelia sinensis*		IC_50_ 0.09 ± 0.02 μM	[80]
-	Aqueous extracts of *Morinda lucida*		IC_50_ 2.30 mg/mL	[81]
-	Ethanolic extract from *Senna surattensi*		IC_50_ 123.95 μg/mL	[82]
-	Acetone extracts from *Picralima nitida*		IC_50_ 6.50 mg/mL	[81]
Hydroxytyrosol derivate from the phenolic compound oleuropein	*Olea europea*		IC_50_ 150 μM at 600 μM	[84]
-	Ethanol-free extract of *Abutilon indicum*		IC_50_ 191.64 mcg/mL	[85]
-	Aqueous methanol extracts of *Ononis angustissima*		IC_50_ 2.01 mg/mL	[86]
-	Distilled water extracts of *Ononis angustissima*		IC_50_ 2.52 mg/mL	
-	Root ethanolic extract of *Cissus cornifolia*		IC_50_ 22.75 ± 1.23 μg/mL	[87]
-	Aqueous root extract of *Cissus cornifolia*		IC_50_ 33.70 ± 3.75 μg/mL	
-	Ethanolic extracts (70%) of *Hibiscus sabdariffa*		IC_50_ 41.77 μg/mL	[88]
-	Aqueous ethanolic extract (70% *v*/*v*) of *Capsicum annuum*		58% at 5 mg/mL	[89]
-	Purple flesh potatoes extract		IC_50_ 25 μg/mL	[90]
Cys-Ser-Ser-Val	Protein hydrolysate from *Andrias davidianus*		IC_50_ 13.76 × 10^3^ μg/mL	[78]
Tyr-Ser-Phe-Arg	Protein hydrolysate from *Andrias davidianus*		IC_50_ 10.82 × 10^3^ μg/mL	
Ser-Ala-Ala-Pro	Protein hydrolysate from *Andrias davidianus*		IC_50_ 4.46 × 10^3^ μg/mL	
Pro-Gly-Gly-Pro	Protein hydrolysate from *Andrias davidianus*		IC_50_ 4.23 × 10^3^ μg/mL	
Leu-Gly-Gly-Gly-Asn	Protein hydrolysate from *Andrias davidianus*		IC_50_ 2.86 × 10^3^ μg/mL	
-	Hexane extracts of leaf essential oil of *Juniperus phonicea*		IC_50_ 30.15 μg/mL	[25]
-	Methanol extract of *Hibiscus sabdariffa*		IC_50_ 29.3 ± 0.5 μg/mL	[34]
-	Aqueous extract of *Tamarindus indica*		IC_50_ 139.4 ± 9 μg/mL	
-	Aqueous extract of *Aframomum danielli*, *Hypodapnis zenkeri, Echinops giganteus*, *Aframomum citratum*, *Xylopia aethiopica*		>75% inhibition	[35]
Polyphenols	Polyphenol rich extracts of *Phaseolus vulgaris*		IC_50_ values ranged from 69 ± 1.9 to 126 ± 3.2 μg/mL and from 107.01 ± 4.5 to 184.20 ± 5.7 μg/mL before and after cooking	[38]
(S)-malic acid	Ethanol and methanol extracts from *Flacourtia inermis*		IC_50_ from 1021 to 1949 ppm	[40]
Syringic acid, *o*-coumaric acid and quercetin	Aqueous extract of *Psidium guajava*		before the exposure to gastric fluid 14410.60 ± 38 inhibited enzyme unit in μmol·min^−1^·g^−1^	[43]
-	*Argyranthemum pinnatifidum* (leaves)		IC_50_ 1.55 ± 0.04 mg/mL	[44]
-	*Argyranthemum pinnatifidum* (flowers)		IC_50_ 2.18 ± 0.05 mg/mL	
-	*Artemisia argentea* (leaves)		IC_50_ 1.81 ± 0.03 mg/mL	
-	*Artemisia argentea* (flowers)		IC_50_ 2.48 ± 0.04 mg/mL	
-	*Helichrysum devium* (leaves)		IC_50_ 1.85 ± 0.06 mg/mL	
-	*Helichrysum devium*		IC_50_ 2.39 ± 0.03 mg/mL,	
-	*Helichrysum melaleucum* (leaves)		IC_50_ 1.71 ± 0.02 mg/mL	
-	*Helichrysum melaleucum* (flowers)		IC_50_ 2.15 ± 0.05 mg/mL	
-	Ethyl acetate extracts pits from Tunisian date palm variety Kentichi		IC_50_ 25.4 ± 0.6 μg/mL	[45]
-	Methanol extracts pits from Tunisian date palm variety Kentichi		IC_50_ 0.072 ± 0.003 μg/mL	
-	Ethanol extract of fresh *Euchema denticulatum*		88% inhibition	[50]
	*Saccharina japonica* fermented by *Monascus purpureus*		IC_50_ 0.98 ± 0.10 mg/mL	[53]

**Table 5:** Natural alpha-glucosidase inhibitors.

Compound	Source	Method	Results	Reference
2,4,6-tribromophenol	Purified from *Grateloupia elliptica*	In vitro	IC_50_ 60.3 μM	[92]
2,4-dibromophenol	Purified from *Grateloupia elliptica*		IC_50_ 110.4 μM	
diphlorethohydroxycarmalol (DPHC)	*Ishige okamure*		IC_50_ 0.16 mM	[83]
-	*Cladophora rupestris*		Methanol (67%) and ethyl acetate (61%) extracts	[94]
-	*Ascophyllum sp.*		IC_50_ ≈ 19 μg/mL	[95]
-	Aqueous extract of *Ulva reticulata*		76.02 ± 0.83% inhibition after 8 h of extraction	[96]
-	Aqueous extract of *Gracilaria edulis*		79.55 ± 3.08% inhibition after 8 h of extraction	
-	Grape seed extract		IC_50_ 1.2 ± 0.2 μg/mL	[79]
-	Green tea extract		IC_50_ 0.5 ± 0.1 μg/mL	
-	White tea extract		IC_50_ 2.5 ± 0.4 μg/mL	
Kaempferol monoglycoside	*Camelia sinensis*		IC_50_ 40.02 ± 4.61 μM	[80]
-	Aqueous extracts of *Morinda lucida*		IC_50_ 2.00 mg/mL	[81]
-	Acetone extracts from *Picralima nitida*		IC_50_ 3.00 μg/mL	
-	Ethanol-free extract of *Abutilon indicum*		IC_50_ = 207.13 mcg/mL	[85]
-	Aqueous methanol extracts of *Ononis angustissima*		IC_50_ 0.94 mg/mL	[86]
-	N-butanol extracts of *Ononis angustissima*		IC_50_ 0.99 mg/mL	
-	Root ethanolic extract of *Cissus cornifolia*		IC_50_ 2.81 ± 0.97 μg/mL	[87]
-	Aqueous root extract of *Cissus cornifolia*		IC_50_ 37.48 ± 2.35 μg/mL	
-	Ethanolic extracts (70%) of *Hibiscus sabdariffa*		IC_50_ 18.09 μg/mL	[88]
-	Aqueous ethanolic extract (70% *v*/*v*) of *Capsicum annuum*		66% at 5 mg/mL	[89]
-	Purple flesh potatoes extract		IC_50_ 42 μg/mL	[90]
Cys-Ser-Ser-Val	Protein hydrolysate from *Andrias davidianus*		IC_50_ 206 μg/mL	[78]
Tyr-Ser-Phe-Arg			IC_50_ 162 μg/mL	
Ser-Ala-Ala-Pro			IC_50_ 66.90 μg/mL	
Pro-Gly-Gly-Pro			IC_50_ 63.50 μg/mL	
Leu-Gly-Gly-Gly-Asn			IC_50_ 42.93 μg/mL	
5-methoxy-7-hydroxy-9,10-dihydro-1,4-phenanthrenequinone	Methanol extract of *Dendobium formosum*		IC_50_ 126.88 ± 0.66μM	[24]
Polyphenols	Polyphenol rich extracts of *Phaseolus vulgaris*		IC_50_ values ranged from 39.3 ± 4.4 to 74.13 ± 6.9 μg/mL and from 51 ± 7.7 to 122.1 ± 5.2 μg/mL before and after cooking	[38]
(S)-malic acid	Ethanol and methanol extracts from *Flacourtia inermis*		IC_50_ from 549 to 710 ppm	[40]
Catechin	Aqueous extract of *Psidium guajava*		Before gastric fluid exposure 28.82 ± 0.02 inhibited enzyme unit in μmol·min^−1^·g^−1^ and after exposure 2.59 ± 0.06 inhibited enzyme unit in μmol·min^−1^·g^−1^	[43]
-	*Argyranthemum pinnatifidum* (leaves)		IC_50_ 0.57 ± 0.03 mg/mL	[44]
-	*Argyranthemum pinnatifidum* (flowers)		IC_50_ 0.81 ± 0.02 mg/mL	
-	*Saccharina japonica* fermented by *Monascus purpureus*		Maltose IC_50_ 0.02 ± 0.07 mg/mL and sucrose IC_50_ 0.08 ± 0.13 mg/mL	[53]
Polyphenols	Winemaking generates by-products		From 75.6 ± 2.5% to 93.7 ± 0.5%, samples treated with pronase and from 84.5 ± 0.5% to 96.5 ± 2.9% viscozyme	[48]

## 4. Conclusions

T2DM is a serious worldwide disease, occuring mainly because of an unhealthy lifestyle. Its prevention and control are achieved with changes in lifestyle, sometimes coupled with medication with several side effects and high costs associated. As a result, the search for new possible anti-hyperglycemic and anti-diabetic agents from natural sources without, or with less, side effects and at a low cost for the patient, has attracted interest from the scientific community, as shown in this review. The natural sources include extracts from plants, fruits, vegetables and specially from marine macro or microorganisms with bioactive compounds. Indeed, marine organisms such as seaweed from edible species have several compounds with modes of action involving specific mechanisms that can be employed in T2DM treatment. The bioactive compounds include polysaccharides and dietary fibers, fatty acids (MUFA and PUFA), and phenolic compounds. Taking into account that the development of T2DM takes time, the glycemic control can be prevented with the intake of healthy foods like seaweed for glycemic control as preventive measures. However, further research in the future is required to fully understand the anti-diabetic mechanisms of this type of food in the prevention and management of this pathology.

## Figures and Tables

**Table 1:** Natural lipase inhibitors.

Compound	Source	Method	Results	Reference
-	Crude extracts of *Rubi Fructus*	In vitro	32.5% inhibition at 100 μg/mL	[23]
-	Crude extracts of *Corni Fructus*		34.8% inhibition at 100 μg/mL	
-	Crude extracts of *Salicis Radicis et Cortex*		38% inhibition at 100 μg/mL	
-	Crude extracts of *Geranium nepalense*		31.4% inhibition at 100 μg/mL	
5-methoxy-7-hydroxy-9,10-dihydro-1,4-phenanthrenequinone	Methanol extract of *Dendobium formosum*		IC_50_ 69.45 ± 10.14 μM	[24]
-	Methanol extracts of leaf essential oil of *Juniperus phonicea*		IC_50_ 60.22 μg/mL	[25]
-	Ethanolic extracts of *Chrysophyllum cainito*		74.91% inhibition and 92.11% for bioactive compounds concentrated in hexane	[26]
-	Methanol extracts of fermented *Camellia japonica*		IC_50_ 0.308 mg/mL	[27]
-	Ethanol extracts of *Camelia sinensis*		IC_50_ 0.5 mg/mL	[28]
(−)-Epigallocatechin-3-gallate	Green Tea *Camelia sinensis*		IC_50_ 7.5 μmol/L	[29]
Theaflavin-3,3′-digallate	Black Tea *Camelia sinensis*		IC_50_ 1.9 μmol/L	
theaflavin-3′-gallate	Black Tea *Camelia sinensis*		IC_50_ 4.2 μmol/L	
theaflavin-3-gallate (Figure 1a)	Black Tea *Camelia sinensis*		IC_50_ 3 μmol/L	
theaflavin	Black Tea *Camelia sinensis*		IC_50_ >10 μmol/L	
(−)-epigallocatechin-3-*O*-gallate	Oolong tea *Camelia sinensis*		IC_50_ 54.97 ± 1.32 μM	[30]
(−)-gallocatechin-3-*O*-gallate	Oolong tea *Camelia sinensis*		IC_50_ 54.68 ± 2.23 μM	
(−)-epicatechin-3-*O*-gallate	Oolong tea *Camelia sinensis*		IC_50_ 38.22 ± 0.89 μM	
Tea saponin	-		EC_50_ 242 mM	[31]
Oleanolic acid	-		EC_50_ 0.895 mM	
Betulin	-		EC_50_ 0.958 mM	
Ginsenoside Ro	-		EC_50_ 0.885 mM	
Ginsenoside Rd	-		EC_50_ 388 mM	
-	Ethanol extracts of *Ceratonia siliqua*		IC_50_ 0.8 mg/mL	[28]
-	Ethanol extracts of *Curcuma longa*		IC_50_ 0.8 mg/mL	
-	Ethanol extracts of *Sarcopoterium spinosum*		IC_50_ 1.2 mg/mL	
-	Ethanol extracts of *Mentha spicata*		IC_50_ 1.2 mg/mL	
-	Crude extract of *Oxalis cordata*		IC_50_ 0.84 mg/mL	[32]
-	Ethyl acetate extract of *Oxalis cordata*		IC_50_ 0.88 mg/mL	
-	Aqueous fraction of *Oxalis cordata*		IC_50_ 0.63 mg/mL	
Phenolic compounds	Methanol extracts of *Eucalyptus globulus*		78.92% inhibition against lipase from *Aspergillus niger* and 82.65% against lipase from olive	[33]
Phenolic compounds	Methanol extracts of *Mentha viridis*		72.75% inhibition against lipase from *Aspergillus niger* and 75.75% against lipase from olive	
-	Methanol extract of *Hibiscus sabdariffa* L.		IC_50_ 35.8 ± 0.8 μg/mL	[34]
-	Methanol extract of *Tamarindus indica* L.		IC_50_ 152 ± 7 μg/mL	
-	Aqueous extracts of *Xylopia aethiopica*		92.25% inhibition	[35]
-	Aqueous extracts of *Scorodophloeus zenkeri*		56.39% inhibition	
Hexadecanoic acid, methyl hexadecanoate, isopropryl palmitate, methyl 9,12-octadecadienate and methyl 9,12,15-octadecatrienoate	Chloroform fractions of *Lagenaria siceraria*		IC_50_ 157.59 μg/mL	[36]
Furoic acid	Compounds derived from the oxidation of vitamin C		IC_50_ 2.12 ± 0.04 mM	[37]
Oxalic acid	-		IC_50_ 15.05 ± 0.78 mM	
Polyphenols	Polyphenol rich extracts of *Phaseolus vulgaris*		IC_50_ values ranged from 63.11 ± 7.5 to 103.2 ± 5.9 μg/mL and from 92 ± 6.3 to 128.5 ± 7.4 μg/mL before and after cooking	[38]
-	Polyphenol rich extracts of *Ascophyllum nodosum*	oil-based turbidimetric assay	IC_50_ 200 μg gallic acid equivalents per assay	[39]
Purified phlorotannin-enriched fraction	Polyphenol rich extracts of *Ascophyllum nodosum*		IC_50_ 60 μg gallic acid equivalents per assay	
(S)-malic acid	Ethanol and methanol extracts of *Lovi* from *Flacourtia inermis*	In vitro	IC_50_ from 1290 to 2096 ppm	[40]
Catechin-like flavan-3-ols	*Acacia mearnsii*		IC_50_ 0.95 mg/mL	[41]
-	Water extract of *Juglans mandshurica*		IC_50_ 2.3 mg/mL	[42]
1,4,8-trihydroxynaphthalene-1-*O*-β-D [6′-*O*-(3′’,4′’,5′’-trihydroxybenzoyl)] glucopyranoside	Water extract of *Juglans mandshurica*		88% inhibition at 1mM	
Epigallocatechin gallate and revesterol	Aqueous extract of *Psidium guajava* (Pedro Sato cultivar)		Before exposure to gastric fluid 36.45 ± 0.68 inhibited enzyme unit in μmol·min^−1^·g^−1^ and after exposure 43.33 ± 1.80 inhibited enzyme unit in μmol·min^−1^·g^−1^	[43]
-	*Phagnalon lowei*		IC_50_ 3.05 ± 0.07 mg/mL	[44]
-	Ethyl acetate extracts pits from Tunisian date palm variety Kentichi		IC_50_ 5.4 ± 0.6 μg/mL	[45]
-	Methanol extracts pits from Tunisian date palm variety Kentichi		IC_50_ 1.21 ± 0.23 μg/mL	
3-*O*-trans-p-coumaroyl actinidic acid	Roots of *Actinidia arguta*		IC_50_ 14.95 μM	[46]
Baicalin	*Scutellaria baicalensis*		IC_50_ 229.22 ± 12.67 μM	[47]
Wogonin	*Scutellaria baicalensis*		IC_50_ 153.71 ± 9.21 μM	
Oroxylin A	*Scutellaria baicalensis*		IC_50_ 56.07 ± 4.90 μM	
Polyphenols	Winemaking generates by-products		From 35.2 ± 0.2% to 45.5 ± 1.2%, samples treated with pronase and from 86.2 ± 0.3% to 94.3 ± 1.5% viscozyme	[48]
-	Grape extract		IC_50_ 8.6 ± 1.1mg/mL	[49]
-	Ethanol extract of dried *Kappaphycus striatus*		92% inhibition	[50]
Caulerpenyne	Ethyl acetate extract of Caulerpa taxifolia		50% inhibition	[51]
Fucofuroeckol A (Figure 1b)	Methanol extract of *Eisenia bicyclis*		IC_50_ 37.2 ± 2.3 μM	[52]
7-phloroeckol	Methanol extract of *Eisenia bicyclis*		IC_50_ 12.7 ± 1 μM	
-	*Saccharina japonica* fermented by *Monascus purpureus*		IC_50_ 4.98 ± 0.85 μg/mL	[53]
Carnosic acid	Methanol extract of *Salvia officialis*		IC_50_ 12 μg/mL at 36 μM	[54]
Carnosol	Methanol extract of *Salvia officialis*		IC_50_ 4.4 μg/mL	
Roylenoic acid	Methanol extract of *Salvia officialis*		IC_50_ 35 μg/mL	
7-methoxyrosmanol	Methanol extract of *Salvia officialis*		IC_50_ 32 μg/mL	
Triterpene oleanolic acid (Figure 1c)	Methanol extract of *Salvia officialis*		IC_50_ 83 μg/mL	
Crocin	*Gardenia jasminoids*		IC_50_ 28.63 μmol	
Lipstatin	*Streptomyces toxytricini*		IC_50_ 0.14 μm	
Panclicins A (Figure 1d)	*Streptomyces* sp.		IC_50_2.9 μM	
Panclicins B	*Streptomyces* sp.		IC_50_ 2.6 μM	
Panclicins C	*Streptomyces* sp.		IC_50_ 0.62 μM	
Panclicins D	*Streptomyces* sp.		IC_50_ 0.66 μM	
Panclicins E	*Streptomyces* sp.		IC_50_ 0.89 μM	
Valilactone	*Streptomyces albolongus*		IC_50_ 0.14 nm	
Ebelactones A	*Streptomyces aburaviensis*		IC_50_ 3 nm/mL	
Ebelactones B	*Streptomyces aburaviensis*		IC_50_ 0.8 nm/mL	
Esterastin	*Streptomyces lavendulae*		IC_50_ 0.2 ng/mL	
Vibralactone	*Boreostereum virans*		IC_50_ 0.14 μg/mL	
Percyquinnin (Figure 1e)	*Basidiomycete stereum complicatum*		IC_50_ 2 μm	

**Table 2:** Natural PTP1B inhibitors.

Compound	Source	Method	Results	Reference
-	Methanol extract from Rosemary	In vitro	IC_50_ 40.9 ± 7.2 μM (commercial)	[57]
-	Methanol extract from Mexican oregano		IC_50_ 37.3 ± 6.8 μM (commercial)	
-	Methanol extract from Marjoram		IC_50_ 32.4 ± 17.5 μM (commercial)	
Mulberrofurans C	*Morus alba*		IC_50_ 0.72 ± 0.09 μM	[59]
Mulberrofurans J (Figure 1f)	*Morus alba*		IC_50_ 0.60 ± 0.07 μM	
Mulberrofurans F	*Morus alba*		IC_50_ 0.57 ± 0.16 μM	
Caffeoylquinic acid derivate chlorogenic acid	Leaves of *Artemisia princeps*		IC_50_ 11.1 μM	[60]
Isoderrone (Figure 1g)	Ethanol-water extract of *Ficus racemosa*		IC_50_ 22.7 ± 1.7 μM	[61]
Derrone	Ethanol-water extract of *Ficus racemosa*		IC_50_ 12.6 ± 1.6 μM	
Alpinumisoflavone	Ethanol-water extract of *Ficus racemosa*		IC_50_ 21.2 ± 3.8 μM	
Mucusisoflavone B	Ethanol-water extract of *Ficus racemosa*		IC_50_ 2.5 ± 0.2 μM	
Sulfircin	*Ircinia*		-	[62]
2,2′,3,3′-Tetrabromo-4,4′,5,5′-tetra-hydroxydiphenyl methane	*Rhodomela confervoides*		IC_50_ 2.4 μM	
3-Bromo-4,5-Bis-(2,3-dibromo- 4,5-dihydroxybenzyl) pyrocatechol (Figure 1h)	*Rhodomela confervoides*		IC_50_ 1.7 μM	
Bis-(2,3-dibromo-4,5-dihydroxybenzyl) ether	*Rhodomela confervoides*		IC_50_ 1.5 μM	
2,2′,3,3′-Tetrabromo-3′,4,4′,5-tetrahydroxy-6′-ethyloxymethyldiphenylmethane	*Rhodomela confervoides*		IC_50_ 0.8 μM	
3,4-Dibromo-5-(2-bromo-3,4-dihydroxy-6-(ethoxymethyl)benzyl)benzene-1,2-diol	*Rhodomela confervoides*		IC_50_ 0.8 μM	
3,4-Dibromo-5-(methoxymethyl)benzene-1,2-diol	*Rhodomela confervoides*		IC_50_ 3.4 μM	
3-(2,3-Dibromo-4,5-dihydroxyphenyl)-2-methylpropanal	*Rhodomela confervoides*		IC_50_ 4.5 μM	
3,4-Dibromo-5-(2-bromo-3,4-dihydroxy-6-(isobutoxymethyl)benzyl)benzene-1,2-diol	*Rhodomela confervoides*		IC_50_ 2.4 μM	
7-Bromo-1-(2,3-dibromo-4,5-dihydroxy phenyl)- 2,3-dihydro-1H-indene-5,6-diol	*Rhodomela confervoides*		IC_50_ 2.8 μM	
2,5,8-Tribromo-3-bromoamino-7-bromomethylnaphthalene	*Laurencia similis*		IC_50_ 65.3 μM	
2,5,6-Tribromo-3-bromoamino-7-bromomethylnaphthalene	*Laurencia similis*		IC_50_ 69.8 μM	
2′,5′,6′,5,6-Pentabromo-3′,4′,3,4-tetramethoxybenzo-phenone	*Laurencia similis*		IC_50_ 2.7 μM	
2-(3′,5′-Dibromo-2′-methoxyphenoxy)-3,5-dibromophenol	*Lamellodysidea herbacea*		IC_50_ 0.9 μM	
3,5-Dibromo-2-(3′,5′-dibromo-2′-methoxyphenoxy)-1-methoxybenzene	*Lamellodysidea herbacea*		IC_50_ 1.7 μM	
3,5-Dibromo-2-(3′,5′-dibromo-2′ -methoxyphenoxy) phenylethanoate	*Lamellodysidea herbacea*		IC_50_ 0.6 μM	
3,5-Dibromo-2-(3′,5′-dibromo-2′-methoxyphenoxy) phenylbutanoate	*Lamellodysidea herbacea*		IC_50_ 0.7 μM	
3,5-Dibromo-2-(3′,5′-dibromo-2′ -methoxyphenoxy) phenylhexanoate	*Lamellodysidea herbacea*		IC_50_ 0.7 μM	
3,5-Dibromo-2-(3′,5′-dibromo-2′-methoxyphenoxy) phenyl benzoate	*Lamellodysidea herbacea*		IC_50_ 1 μM	
5,5′-(3-Bromo-4,5-dihydroxy-1,2-phenylene)-Bis-(methylene))Bis-(3,4-dibromobenzene-1,2-diol)	*Rhodomela confervoides*		IC_50_ 1.7 μM	
3,4-Dibromo-5-(2-bromo-3,4-dihydroxy-6-(ethoxymethyl)benzyl)benzene-1,2-diol	*Rhodomela confervoides*		IC_50_ 0.84 μM	
2-(30,50-Dibromo-20-methoxyphenoxy)-3,5-dibromophenol	*Lamellodysidea herbacea*		IC_50_ 0.9 μM	
2-(30,50-Dibromo-20-methoxyphenoxy)-3,5-dibromophenol-methyl ether	*Lamellodysidea herbacea*		IC_50_ 1.7 μM	
2,3,6-Tribromo-4,5-dihydroxybenzyl methyl ether	*Symphyocladia latiuscula*		IC_50_ 3.9 μM	
Bis-(2,3,6-tribromo-4,5-dihydroxyphenyl) methane	*Symphyocladia latiuscula*		IC_50_ 4.3 μM	
1,2-Bis-(2,3,6-tribromo-4,5-dihydroxyphenyl)-ethane	*Symphyocladia latiuscula*		IC_50_ 2.7 μM	
3′,5′,6′,6-Tetrabromo-2,4-dimethyldiphenyl ether	*Laurencia similis*		IC_50_ 3 μM	
2′,5′,6′,5,6-Pentabromo-3′,4′,3,4-tetramethoxybenzo-phenone	*Laurencia similis*		IC_50_ 2.7 μM	
3′,5′,6′6-Tetrabromo-2,4-dimethyldiphenyl ether	*Laurencia similis*		IC_50_ 3 μM	
1,2,5-Tribromo-3-bromoamino-7-bromomethylnaphthalene	*Laurencia similis*		IC_50_ 102 μM	

**Table 3:** Natural DPP4 inhibitors.

Compound	Source	Method	Results	Reference
Caseins	Protein hydrolysates cow’s milk	In silico	Occurrence frequency 0.249 (beta-casein), 0.380 (bovine meat) and 0.305 (salmon). Gly-Ala, Gly-Pro and Pro-Gly were the most frequently occurring sequences	[69]
Collagens	Protein hydrolysates bovine meat and salmon			
Gly-Pro-Gly-Ala	Protein hydrolysates Atlantic salmon skin gelatin	In vitro	IC_50_ 49.6 μM	[66]
Gly-Pro-Ala-Glu			IC_50_ 41.9 μM	
Pro-Gly-Val-Gly-Gly-Pro-Leu-Gly-Pro-Ile-Gly-Pro-Cys-Tyr	Protein hydrolysates tuna cooking juice		IC_50_ 116 μM	
Cys-Ala-Tyr-Gln-Trp-Gln-Arg-Pro-Val-Asp-Arg-Ile-Arg			IC_50_ 78 μM	
Pro-Ala-Cys-Gly-Gly-Phe-Tyr-Ile-Ser-Gly-Arg-Pro-Gly			IC_50_ 96.4 μM	
Leu-Pro	Protein hydrolysates Japanese rice bran		IC_50_ 2400 μM	
Ile-Pro			IC_50_ 410 μM	
Met-Pro			IC_50_ 870 μM	
Val-Pro			IC_50_ 880 μM	
Arg-Pro			IC_50_ 2240 μM	
Thr-Pro			IC_50_ 2370 μM	
Leu-Pro			IC_50_ 2370 μM	
Lys-Pro	Protein hydrolysates Japanese rice bran		IC_50_ 2540 μM	
His-Pro			IC_50_ 2820 μM	
Tyr-Pro			IC_50_ 3170μM	
Phe-Pro			IC_50_ 3630 μM	
Trp-Pro			IC_50_ 4530 μM	
Pro-Pro			IC_50_ 5860 μM	
Ser-Pro			IC_50_ 5980 μM	
Ala-Pro			IC_50_ 7950 μM	
Leu-Pro-Gln-Asn-Ile-Pro-Pro-Leu	Protein hydrolysates gouda cheese		IC_50_ 46 μM	
Leu-Pro-Gln-Asn-Ile-Pro-Pro			IC_50_ 160μM	
Pro-Gln-Asn-Ile-Pro-Pro-Leu			IC_50_ 1500 μM	
Leu-Pro-Gln			IC_50_ 82 μM	
Val-Pro-Ile-Thr-Pro-Thr			IC_50_ 130 μM	
Val-Pro-Ile-Thr-Pro-Thr-Leu			IC_50_ 110 μM	
Phe-Pro-Gly-Pro-Ile-Pro-Asp			IC_50_ 260 μM	
Pro-Gly-Pro-Ile-His-Asp-Ser			IC_50_ 1000 μM	
Ile-Pro-Pro-Leu-The-Gln-Thr-Pro-Val			IC_50_ 1300 μM	
Val-Pro-Pro-Phe-Ile-Gln-Pro-Glu			IC_50_ 2500 μM	
Tyr-Pro-Phe-Pro-Gly-Pro-Ile-Pro-Asp			IC_50_ 670 μM	
Val-Ala-Gly-Thr-Trp-Tyr	Protein hydrolysates β-lactoglobulin		IC_50_ 174 μM	
Ile-Pro-Ala			IC_50_ 49 μM	
Ile-Pro-Ala-Val-Phe			IC_50_ 45 μM	
Ile-Pro-Ala-Val-Phe-Lys			IC_50_ 143 μM	
Val-Leu-Val-Leu-Asp-Thr-Asp-Tyr-Lys			IC_50_ 424 μM	
Thr-Pro-Glu-Val-Asp-Asp-Glu-Ala-Leu-Glu-Lys			IC_50_ 320 μM	
Glu-Lys	Protein hydrolysates milk protein		IC_50_ 3216 μM	
Gly-Leu			IC_50_ 2615 μM	
Ala-Leu			IC_50_ 882 μM	
Val-Ala			IC_50_ 168 μM	
Trp-Val			IC_50_ 65 μM	
Phe-Leu			IC_50_ 399 μM	
His-Leu			IC_50_ 143 μM	
Ser-Leu			IC_50_ 2517 μM	
Trp-Val	Synthetic dipeptides		IC_50_ 0.020 ± 0.001 mg/mL	[70]
Lactoferrin hydrolysate LFH1	Milk		IC_50_ 1.088 ± 0.106 mg/mL	
Casein hydrolysate CasH2			IC_50_ 0.882 ± 0.057 mg/mL	
Cirsimaritin, naringenin, hispidulin, eriodictyol and carnosol	Methanol extract of Rosemary		IC_50_ 28.7 ± 3.1 μM (greenhouse-grown) 6.5 ± 0.4 μM (commercial)	[57]
	Methanol extract of Mexican oregano		IC_50_ 25.3 ± 0.3 μM (greenhouse-grown) 3.9 ± 0.6 μM (commercial)	
-	Methanol extract of Marjoram		IC_50_ 37.7 ± 7.9 μM (greenhouse-grown)	
Cirsimaritin	Purified compounds		IC_50_ 0.43 ± 0.07 μM	
Hispidulin			IC_50_ 0.49 ± 0.06 μM	
Naringenin			IC_50_ 2.5 ± 0.29 μM	
Anthocyanins	Blueberry-blackberry wine blends		IC_50_ 0.07 ± 0.02 to >300 μM	[71]
Resveratrol (Figure 1i)	Commonly found in citrus, berry, grape and soybean		IC_50_ 0.6 ± 0.4 nM	
Luteolin			IC_50_ 0.12 ± 0.01 μM	
Apigenin			IC_50_ 0.14 ± 0.02 μM	
Flavone (Figure 1j)			IC_50_ 0.17 ± 0.01 μM	
-	Ethanolic precipitate of *Sargassum binderi*		IC_50_ 2.194 mg/mL	[64]
-	Ethanolic precipitate of *Padina sulcata*		IC_50_ 2.306 mg/mL	
-	Ethanolic precipitate of *Turbinaria conoides*		IC_50_ 3.594 mg/mL	
-	Methanol extract of *Sargassum wightii*		IC_50_ 38.27 μg/mL	[65]
-	Methanol extract of *Sargassum polycystum*		IC_50_ 36.94 μg/mL	
-	Methanol extract of *Turbinaria conoides*		IC_50_ 55.2 μg/mL	[72]
Mangiferin	*Mangifera indica*	Rat model and ELISA	89 ± 8%	[68]
-	Crude extract of marine sponge *Xetospongia muta*	In vitro	IC_50_ 0.8 mg/mL (preincubation time: 10 min) Treatment with 2.5% TCA or heat (60 °C) increase 3.7 and 2.7 total inhibitory activity	[73]
-	Crude extracts of marine anemone *Bunodosoma granulifera*		IC_50_ 1.2 mg/mL (preincubation time: 10 min) thermal treatment destroyed inhibitory activity	
-	Crude extracts of marine anemone *Bartholomea annulata*		IC_50_ 0.38 mg/mL (preincubation time: 3 min) heat or TCA treatment decreased inhibitory activity	
